# Elderly patients undergoing mechanical ventilation in and out of intensive care units: a comparative, prospective study of 579 ventilations

**DOI:** 10.1186/cc8935

**Published:** 2010-03-30

**Authors:** David Lieberman, Liat Nachshon, Oleg Miloslavsky, Valery Dvorkin, Avi Shimoni, Julian Zelinger, Michael Friger, Devora Lieberman

**Affiliations:** 1 The Pulmonary Unit, The Soroka University Medical Center, Beer-Sheva 84101, Israel; 2 The Division of Internal Medicine, The Soroka University Medical Center, Beer-Sheva 84101, Israel; 3 Faculty of Health Sciences, Ben-Gurion University of the Negev, Beer-Sheva 84105, Israel; 4 Hospital Administration, The Soroka University Medical Center, Beer-Sheva 84101, Israel; 5 Department of Epidemiology, Faculty of Health Sciences, Ben-Gurion University of the Negev, Beer-Sheva 84105, Israel; 6 Department of Geriatric Medicine, The Soroka University Medical Center, Beer-Sheva 84101, Israel

## Abstract

**Introduction:**

Many mechanically ventilated elderly patients in Israel are treated outside of intensive care units (ICUs). The decision as to whether these patients should be treated in ICUs is reached without clear guidelines. We therefore conducted a study with the aim of identifying triage criteria and factors associated with in-hospital mortality in this population.

**Methods:**

All mechanically invasive ventilated elderly (65+) medical patients in the hospital were included in a prospective, non-interventional, observational study.

**Results:**

Of the 579 ventilations, 283 (48.9%) were done in ICUs compared with 296 (51.1%) in non-ICU wards. The percentage of ICU ventilations in the 65 to 74, 75 to 84, and 85+ age groups was 62%, 45%, and 23%, respectively. The decision to ventilate in ICUs was significantly and independently influenced by age (Odds Ratio (OR) = 0.945, *P *< 0.001), and pre-hospitalization functional status by functional independence measure (FIM) scale (OR = 1.054, *P *< 0.001). In-hospital mortality was 53.0% in ICUs compared with 68.2% in non-ICU wards (*P *< 0.001), but the rate was not independently and significantly affected by hospitalization in ICUs.

**Conclusions:**

In Israel, most elderly patients are ventilated outside ICUs and the percentage of ICU ventilations decreases as age increases. In our study groups, the lower mortality among elderly patients ventilated in ICUs is related to patient characteristics and not to their treatment in ICUs *per se*. Although the milieu in which this study was conducted is uncommon today in the western world, its findings point to possible means of managing future situations in which the demand for mechanical ventilation of elderly patients exceeds the supply of intensive care beds. Moreover, the findings of this study can contribute to the search for ways to reduce costs without having a negative effect on outcome in ventilated elderly patients.

## Introduction

Mechanical ventilation is the highest priority indication for admission to ICUs according to accepted guidelines [1]. In Israel the shortage of ICU beds, taken together with the growing number of patients who need them, has led to a state in which the threshold for ICU-refusal for ventilated elderly patients is much lower than might be expected in accordance with the consensus statement [2]. As a result, a significant percentage of ventilated elderly patients are treated outside the ICU. This reality, which is very common in Israel but much less so in the rest of the western world is, as would be expected, not well reported in the literature. The vast majority of series dealing with mechanical ventilation primarily addresses patients in ICUs [3-21], and only a few papers also describe patients ventilated outside these units [22,23]. In a comprehensive review of the literature we did not find a single study that included all ventilated elderly patients and compared those treated in ICUs with those who were not. The present study was designed to address this deficiency in the literature.

The aims of this prospective study were: (a) to measure the extent of mechanical ventilation in ICUs compared to non-ICU wards among all elderly patients who were ventilated for medical reasons; (b) to determine which characteristics affect the decision to admit ventilated elderly patients to the ICU; and (c) to determine the factors that affect in-hospital mortality in the combined population and whether admission to the ICU is one of those factors.

## Materials and methods

### Study population

All hospitalized patients 65 years or older who underwent tracheal intubation for mechanical ventilation during the study period for reasons unrelated to trauma and/or surgical intervention, were included in the study. Stroke patients ventilated due to respiratory failure were excluded. The patients were ventilated in seven internal medicine wards (320 beds in all), in the general ICU (medical and surgical, 12 beds in all), in the medicine ICU (eight beds), and in the intensive coronary care unit (ICCU, seven beds) in the Soroka University Medical Center in Beer-Sheva, a 1,100 bed tertiary hospital in southern Israel. Patients who had a permanent tracheostomy were included in the study only if they breathed spontaneously during the month prior to hospitalization. Patients who underwent tracheal intubation and mechanical ventilation during the course of cardiopulmonary resuscitation were included in the study only if the ventilation continued for more than two hours after the conclusion of the resuscitation. The study staff was not involved in any way in the decision to ventilate the patients or in the decision as to the site (ICU or non-ICU) in which they were ventilated. The study was approved by the Committee for Research in Human Beings (the Helsinki Committee) of the Soroka University Medical Center that waived the need for informed patient consent for this study.

### Study protocol

The study was a prospective, observational, non-interventional survey. Every morning throughout the study period a research staff member went through all the study wards and units and identified patients who began mechanical ventilation the previous day and met the inclusion criteria. For these patients a broad range of data was collected, as detailed below. The data sources were bedside records and patient charts, interviews with the patient's family and/or caregivers, and the computerized patient database system (medical and administrative) in the community and in the hospital. All the data were collected, entered into the study database, and analyzed by a computerized system.

### Collected data

The following data were collected for each of the ventilated patients in the study population: demographic data, the setting from which the patient came to the hospital (community, nursing care), use of home oxygen, previous mechanical ventilation, chronic diseases and their severity as quantified by the Charlson score [24], pre-hospitalization functional status (two weeks before the present hospitalization) by the FIM scale [25], the medical indication for mechanical ventilation, the physiological condition of the patient on the first day of ventilation by APACHE II score [26], and in-hospital mortality.

### Classification of ventilation

For the purposes of this study, ventilation was classified as *ICU ventilation *if at least one of the following three conditions was met: (a) ventilation in an ICU continued for at least 48 hours, (b) the entire period of ventilation took place in an ICU, even if it was less than 48 hours, and/or (c) the patient died while being ventilated in an ICU (unrelated to the amount of ventilation time there). Any ventilation that did not meet at least one of these three conditions was classified as *non-ICU ventilation*. Repeat ventilation during the course of the same hospitalization was considered as the same ventilation. Ventilation during another hospitalization for the same patient, during the course of the study, was considered separate ventilation.

### Non-ICU ventilation set-up

The set-up for ventilation in the internal medicine wards included three to four patients who were treated in the same room under the supervision of a nurse who was trained in the care of patients of this type. The indications for mechanical ventilation, the ventilation technique, and the ventilation machines were identical to those in the ICUs. The patients were under continual electrocardiographic (ECG) monitoring and vital signs were measured every few hours. Central venous lines were inserted when indicated, but arterial lines and Swan-Ganz catheters were not used. The doctors who treated these patients in the internal medicine wards treated 40 to 50 other patients in the ward as well. During regular daytime work hours these patients are attended by four-to-five doctors and during the night by one doctor. All internal medicine doctors undergo training in medical ICUs as part of their professional development and are skilled in the management of mechanically ventilated patients.

### Statistical analyses

All collected data were entered into an EPI-DATA database. Comparison of the variables between ICU and non-ICU ventilations was conducted by the chi-square test or one-way analysis of variance (ANOVA) in accordance with the type of variable.

Multivariate logistic regression models were used to estimate the independent (adjusted) effects of patients' characteristics on the outcomes (hospitalization of a ventilated patient in an ICU and in-hospital mortality of ventilated patients). The models included variables that were found to have a significant association in the univariate analysis as well as those that had clinical significance (listed in the Results section). SPSS (Statistical Package for the Social Sciences, SPSS Inc, Chicago, IL, USA) statistical software (Version 14.0) was used for data processing and statistical analysis. Statistical significance was set at *P *< 0.05 throughout.

## Results

In the course of two years between 1 July 2004 and 30 June 2006, there were 51,723 hospitalizations in the internal medicine wards of the Soroka University Medical Center and 909 ventilations for medical indications (stroke excluded) were recorded in patients aged 18 years or older. Of these ventilations, 330 (36.3%) were of patients 18 to 65 years of age. In accordance with the study definitions 277 (83.9%) of these were ICU ventilations.

The 579 other ventilations were done in 553 elderly patients 65 years or older (20 patients had two ventilations and two patients had three ventilations each in different hospitalizations during the study period, with an interval of at least six months between any two episodes). This group of ventilations comprised the study population. Of these ventilations, 283 (48.9%) were ICU ventilations compared with 296 (51.1%) non-ICU ventilations.

**Figure 1:**
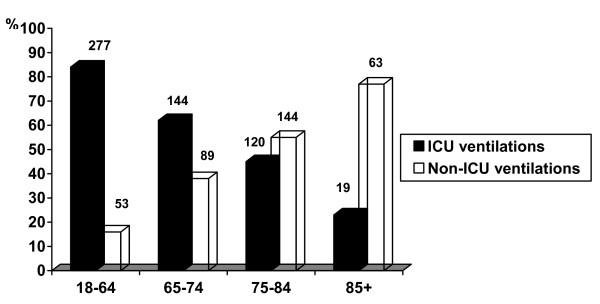
**Comparison of the distribution (%) of ICU vs. non-ICU ventilations, by age group**. The number above each column is the number of ventilations.

Figure 1 presents all 909 ventilations divided between young (18 to 65 years) and elderly patients and into three sub-groups among the elderly patients. These four groups were compared in relation to the percentage of ICU ventilations. The graph demonstrates dramatically that the percentage of ICU ventilations dropped sharply with increasing age.

Of the 296 non-ICU ventilations there was a documented explanation in 172 cases (58.1%) for the decision by an ICU physician not to admit the patient to an ICU. In each of these cases the reason for the decision was either that the patient was not suited for an ICU or that no place was available in an ICU at the time. In the other 124 cases (41.9%) the ward physicians decided not to request transfer to an ICU. The reasons for this decision (obtained by direct questioning by the investigators) were that the case did not justify use of an expensive ICU bed and/or their impression, in light of familiarity with the decision process by ICU physicians, that there was no chance that the patient would be accepted to an ICU.

Table 1 presents a comparison of demographic characteristics and background medical information and the pre-hospitalization functional status for the two study groups. The distribution of the functional status is shown by grouping the FIM score into three functional conditions in addition to the total motor and cognitive FIM scores. In each of these presentation formats there is a conspicuous difference in the functional status between ICU and non-ICU ventilations, in which the latter had a significantly lower pre-hospitalization functional status.

**Table 1:** Comparison of socio-demographic characteristics and medical background between the study sub-groups for elderly patients only

Variable	ICU ventilations N = 283	Non-ICU ventilations N = 296	*P*
Age (years)			
Mean ± SD	75.0 ± 6.3	79.2 ± 7.6	< 0.001
Range	65-99	65-107	

Males (N [%])	124 (43.8)	148 (50.0)	0.09

Charlson score (mean ± SD)	4.1 ± 2.4	4.0 ± 2.5	0.52

Hospitalization from nursing setting (N [%])	4 (1.4)	44 (14.9)	< 0.001

Home oxygen therapy (N [%])	25 (8.8)	49 (16.6)	0.02

Previous mechanical ventilation (N [%])	12 (4.2)	40 (13.5)	< 0.001

			

*Motor FIM (mean ± SD)	73.4 ± 16.7	51.3 ± 24.1	< 0.001

†Cognitive FIM (mean ± SD)	31.5 ± 4.4	24.5 ± 8.8	< 0.001

Total FIM (mean ± SD)	104.9 ± 19.5	75.8 ± 31.2	< 0.001

Distribution of functional status by grouped FIM score:			

Fully dependent - FIM < 60 (N [%])	14 (5)	114 (38)	
Needs a lot of help - 60 ≤ FIM < 90 (N [%])	35 (12)	79 (27)	< 0.001
Needs a little help/independent - FIM ≥ 90 (N [%])	234 (83)	103 (35)	

			

Acute Physiology Score (APS) points (mean ± SD)	13.8 ± 6.4	15.0 ± 5.0	0.02
Age points (mean ± SD)	5.5 ± 0.5	5.6 ± 0.5	< 0.001
Chronic health points (mean ± SD)	1.9 ± 2.4	1.4 ± 2.2	0.06
Total APACHE II score (mean ± SD)	21.2 ± 7.1	22.0 ± 5.8	0.06

*Motor FIM - sum of scores for: self care + sphincter control + mobility + locomotion

†Cognitive FIM - sum of scores for: communication + social cognition

Table 1 also presents a comparison of the Acute Physiology and Chronic Health Evaluation II (APACHE II) scores in the first day of ventilation in the two study populations by total score and by three component sub-scores. The mean total score was higher among the non-ICU ventilations and the difference was very close to statistical significance.

Table 2 presents a comparison of the distribution of diagnoses that led to mechanical ventilation in the two study groups. Significant differences were found for four of seven variables: respiratory insufficiency secondary to sepsis, pulmonary edema, community-acquired pneumonia and cardiogenic shock.

**Table 2:** Comparison of the distribution of the main diagnostic reasons for ventilation in the entire study population and in the two sub-groups (N (%))

Variable	All N = 579	ICU ventilations N = 283	Non-ICU ventilations N = 296	*P*
Respiratory insufficiency secondary to sepsis	169 (29.2)	68 (24.0)	101 (34.1)	0.01

Pulmonary edema	89 (15.4)	53 (18.7)	36 (12.2)	0.04

Community-acquired pneumonia	69 (11.9)	24 (8.4)	45 (15.2)	0.02

Acute exacerbation of COPD	44 (7.6)	21 (7.4)	23 (7.8)	0.99

Respiratory insufficiency following cardiopulmonary arrest	43 (7.4)	22 (7.8)	21 (7.1)	0.88

Hospital-acquired pneumonia	24 (4.1)	11 (3.9)	13 (4.4)	0.92

Cardiogenic shock	17 (2.9)	15 (5.3)	2 (0.7)	< 0.003

Total	455 (78.6)	214 (75.6)	241 (81.4)	

Table 3 presents the results of the multivariate analysis with ICU ventilations as the dependent variable. The predictors in this analysis were age, sex, the Charlson score, hospitalization from a nursing home, use of home oxygen, previous mechanical ventilation, the patient's pre-hospitalization functional status by FIM scale, the Acute Physiological score from the APACHE II score, and the presence and absence of one of seven clinical diagnoses (detailed in Table 2) that were the reason for ventilation. Only the two predictors detailed in the table had a significant and independent effect on the decision to treat the patient in an ICU ward. Both older age and lower functional status had negative effects on the decision. All other predictors including the Acute Physiological score from the APACHE II did not have a significant and independent effect on the decision.

**Table 3:** Results of the regression analysis for the decision to ventilate in the ICU listed by strength of contribution (absolute value of B)

Predictor	Regression coefficient (B)	SE of B	Odd's Ratio (OR)	95% CI of OR	*P*
Age (years)	-0.056	0.011	0.945	(0.924; 0.967)	< 0.001

Pre-hospitalization FIM score (18 to 126)	0.053	0.006	1.054	(1.041; .067)	< 0.001

The number of ventilations that ended in in-hospital mortality among the ICU ventilations was 150 (53.0%) compared to 202 (68.2%) of the non-ICU ventilations (*P *< 0.001). Table 4 presents the results of the multivariate analysis for all ventilated elderly patients with in-hospital mortality as the dependent variable. The predictors in this analysis were those described for the previous multivariate analysis with the addition of ICU ventilation. Only the five predictors listed in the table had a significant and independent effect on in-hospital mortality in the total population. In this case the two most influential factors were conditions that led to ventilation, respiratory insufficiency secondary to sepsis as a positive predictor and pulmonary edema as a negative one. Other independent and significant predictors of in-hospital mortality were more chronic co-morbid conditions assessed by a higher Charlson score, greater physiological impairment assessed by the Acute Physiological score from the APACHE II, and older age. Conspicuously absent from this list was ICU ventilation and pre-hospitalization functional status, which were included in the analysis but were not found to have an independent and significant effect on in-hospital mortality.

**Table 4:** Results of the regression analysis for in-hospital mortality listed by strength of contribution (absolute value of B)

Predictor	Regression coefficient (B)	SE of B	Odds Ratio (OR)	95% CI of OR	*P*
Sepsis as the reason for ventilation (0 = No, 1 = Yes)	0.918	0.295	2.505	(1.405; 4.468)	0.002

Pulmonary edema as the reason for ventilation (0 = No, 1 = Yes)	- 0.746	0.358	0.474	(0.235; 0.957)	0.037

Charlson score (0 to 37)	0.123	0.049	1.131	(1.028; 1.245)	0.011

Acute Physiology Score points (0 to 60)	0.056	0.020	1.058	(1.016; 1.101)	0.006

Age (years)	0.027	0.008	1.027	(1.011; 1.044)	0.001

					

*ICU ventilation (0 = No, 1 = Yes)	-0.186	0.341	0.830	(0.426; 1.619)	0.584

*Pre-hospitalization FIM score (18 to 126)	-0.005	0.004	0.995	(0.987; 1.004)	0.258

*These variables were not statistically significant, but their importance is critical.

## Discussion

This paper focuses on the population of elderly patients who required mechanical ventilation, which in most cases was conducted in a non-ICU setting. This practice is very common in Israel, but less so in other countries in the western world. In this unique reality the question arises as to how generalizable the data and findings of this study are to a non-Israeli setting? In this respect it is noteworthy that there are many hospitals in the world in which, for various reasons, not all elderly patients are ventilated in ICUs. The findings of this study are very relevant for those settings. Furthermore, the combination of increased life expectancy that causes ageing of the population together with a deterioration in the economic state in the western world could lead, in just a few years, to a state in which the demand for mechanical ventilation for elderly patients in an advanced degree of disability exceeds the supply of expensive ICU beds, making the search for new solutions mandatory. The reality in which our study was conducted would, under those circumstances, be much more relevant and could serve as a model for testing ways of dealing with this problem in many countries in the western world. Indeed, our findings can contribute to the search for ways to reduce costs without making the outcome of ventilated elderly patients worse.

The percentage of ICU ventilations among younger patients (18 to 65 years) reached 84%, in contrast to a corresponding rate of only 49% in the elderly group. In addition, in the elderly age group there was a dramatic decrease in this percentage by age. Moreover, in the multivariate analysis of the various predictors of the decision to hospitalize the ventilated elderly in an ICU or not, age was found to have a significant and independent effect. The recommendation that 'chronological age *per se *is not a relevant criterion for hospitalization in an ICU' [27] was not substantiated in the present study population.

Several methodological decisions that were taken in the present study clearly affected its results and require discussion. The decision to study only medical ventilations stemmed from the understanding that this type of ventilation is relatively devoid of non-medical administrative issues that sometimes affect the decision to hospitalize post-operative patients in ICUs. Another critical decision that we took was how to define ICU ventilation for this study. We did not think that the option to define such patients as anyone who was ventilated only in an ICU would be appropriate in light of the high rate of patient transfers from ICUs to wards and vice versa while they are still being ventilated. Under these circumstances we decided to define ICU ventilation as one in which a patient was ventilated in an ICU for a significant and/or a critical portion of the ventilation period. In light of this definition we defined three parameters, any of which would qualify the ventilation as ICU ventilation for purposes of this study.

Another problematic issue was how to relate to patients who were ventilated in wards but were not presented at any time to an ICU staff. In each of these 124 ventilations the background for not presenting the patient to an ICU consultant was the strong feeling of the treating physician that the patient was not suited for an ICU or that the request would be turned down by an ICU consultant. In light of this we decided not to separate these ventilations from those in which the patients were presented to an ICU and were rejected and considered all of them as non-ICU ventilations. In this study we looked at the course of ventilation in elderly patients at two points in time only. One was at the beginning of ventilation when we related to any data that could be collected up to that time. The second point of time was at the end of hospitalization when we related to in-hospital mortality. Relating exclusively to these two points of time was essential so that we could, on the one hand, manage the study objectives, while, on the other, not make the study too cumbersome. In light of this strategy we purposely ignored the course of ventilation and its complications. For the same reason we also related to repeat ventilations in the same hospitalization as one prolonged ventilation.

The *ICU gatekeeper *who has to conduct triage and decide who should and who should not be admitted to an ICU is not equipped with well-defined guidelines for this task. The decision as to whether or not to admit a ventilated patient to an ICU should be reached on the basis of clinical and ethical considerations and in accordance with available space in an ICU at the given time. These considerations are very poorly defined for elderly patients and give the decision maker broad latitude. Thus, the appropriate method to identify the basis for the triage decision is to analyze its results. The univariate analyses of the various variables between the ICU and non-ICU ventilations identified significant differences between these two sub-groups in terms of a broad range of characteristics. From among these predictors the multivariate analyses *filtered *out only two that had a significant and independent effect on the decision to hospitalize the ventilated patient in an ICU. These two influential factors were age, which was discussed above and was also found in a previous study [28], and the pre-hospitalization functional status of the patient. Despite the ethical problems relating to this issue, in practice the triage staff looked at higher age and poor functional status as negative factors in the decision to hospitalize the patient in an ICU. Among the variables that did not pass this *filtering *process the Acute Physiological score points component of the APACHE II score is noteworthy. This reflects a lack of significant consideration of the severity of the elderly patient's condition at the initiation of ventilation among the factors that influenced the decision to hospitalize in an ICU.

The primary importance of the list of variables that affect in-hospital mortality of elderly ventilated patients lies in the two variables that did not affect mortality. The first variable is the baseline functional status of the elderly patient. The explanation for the finding that this variable did not affect in-hospital mortality of elderly ventilated patients is that patients with a low functional status usually also have the characteristics that were found in this analysis to significantly and independently affect mortality, in particular very advanced age, a higher Charlson score, and a greater propensity for sepsis. When these factors are controlled, functional status does not have a significant independent effect on mortality. The second variable, ICU ventilation, did not have a significant independent effect on in-hospital mortality even though it was included in the analyses. One ramification of this finding is that the significantly low rate of in-hospital mortality among ICU ventilations compared to non-ICU ventilations in this study stemmed from the different characteristics of the patients in these two sub-groups and not from hospitalization in an ICU, *per se*. The other significance of this finding requires extra caution. The elderly ventilated population in this study underwent selection into two sub-groups on the basis of actual decisions as to where to hospitalize them. In this population and in accordance with this selection process in-hospital mortality was not affected by ICU ventilations as defined for the study. Despite this finding, it should not be inferred under any circumstances that hospitalization in an ICU does not contribute to the reduction of in-hospital mortality in other populations, using other triage methods and with other definitions of ICU ventilations. Another important aspect of the list of variables that affect in-hospital mortality is in its comparison to the list of factors that affect the decision to hospitalize elderly patients in an ICU. Although age is included in both lists, the other variables are included in only one of them. If survival at the end of the hospital period were the only or primary index for the success of ventilation in the study population, it would be reasonable to expect a greater similarity between the two lists. The striking difference between the two lists reflects, in our opinion, the approach that in elderly ventilated populations, in-hospital mortality is not the only measure and apparently is not even the most important measure of success. Because we feel that this issue of the most appropriate measure of success in the population of ventilated elderly patients is of utmost importance we analyzed it on the same cohort from the perspective of one year after discharge from the hospital. This analysis was published in a separate paper that was dedicated to this issue [29].

## Conclusions

In Israel, most elderly patients are ventilated outside ICUs and the percentage of ICU ventilations decreases as age increases. In our study groups, the lower mortality among elderly patients ventilated in ICUs is related to patient characteristics and not to their treatment in ICUs *per se*. Although the milieu in which this study was conducted is uncommon today in the western world its findings point to possible means of managing future situations in which the demand for mechanical ventilation of elderly patients exceeds the supply of intensive care beds. Moreover, the findings of this study can contribute to the search for ways to reduce costs without having a negative effect on the outcome in ventilated elderly patients.

## Key messages

• In Israel, most elderly patients are ventilated outside ICUs.

• In Israel, the percentage of ICU ventilations decreases as age increases.

• The lower mortality among elderly patients ventilated in ICUs is related to patient characteristics and not to their treatment in ICUs *per se*.

• The findings of this study can contribute to the search for ways to reduce costs without having a negative effect on the outcome in ventilated elderly patients.

## Abbreviations

ANOVA: analysis of variance; APACHE: Acute Physiology and Chronic Health Evaluation; FIM: functional independence measure; ICU: intensive care unit; ICUs: intensive care units; SPSS: Statistical Package for the Social Sciences.

## Competing interests

The authors declare that they have no competing interests.

## Authors' contributions

DaL conceived the study and its design, conducted it, participated in statistical anlyses and participated in all stages of manuscript preparation. LN, OM, VD and AS were involved in conducting the study. JS and MF participated in conceiving and designing the study, in statistical analyses, and in preparation of the manuscript. DeL participated in the conception, design and conduct of the study as well as preparation of the manuscript. All authors read and approved the final manuscript.
